# Targeting Wnt/EZH2/microRNA-708 signaling pathway inhibits neuroendocrine differentiation in prostate cancer

**DOI:** 10.1038/s41420-019-0218-y

**Published:** 2019-09-30

**Authors:** Jingxuan Shan, Mariam A. Al-Muftah, Moza K. Al-Kowari, Sirin W. J. Abuaqel, Khalid Al-Rumaihi, Issam Al-Bozom, Pu Li, Lotfi Chouchane

**Affiliations:** 1 Department of Genetic Medicine, Weill Cornell Medicine, New York, NY 10065 USA; 2 Department of Microbiology and Immunology, Weill Cornell Medicine, New York, NY 10065 USA; 3 Laboratory of Genetic Medicine and Immunology, Weill Cornell Medicine-Qatar, Doha, Qatar; 4 Cancer Research Center, Qatar Biomedical Research Institute, Hamad Bin Khalifa University, Qatar Foundation, Doha, Qatar; 5 Department of Urology, Hamad Medical Corporation, Doha, Qatar; 6 Department of Laboratory Medicine and Pathology, Hamad Medical Corporation, Doha, Qatar; 7 Department of Pediatrics, Ruijin Hospital and Ruijin Hospital North, Shanghai Jiao Tong University School of Medicine, 200025 Shanghai, China

**Keywords:** Prostate cancer, Prostate cancer

## Abstract

Prostate cancer (PC) castration resistance has been linked to the differentiation of PC luminal cells into hormone-refractory neuroendocrine (NE) cells. However, the molecular mechanisms controlling the emergence of lethal NE prostate cancer (NEPC) remain unclear. The present study aimed to investigate the mechanisms underlying the transition from prostate adenocarcinoma to NEPC. The microRNA miR-708 was involved in NE differentiation and was downregulated in NEPC cells and tumor specimens. miR-708 targeted Sestrin-3 to inhibit Forkhead Box O1 (FOXO1) phosphorylation, resulting in apoptosis of prostate adenocarcinoma cells and AKT-inactivated NEPC cells, the latter of which was consistent with the progression of tumor xenografts in mice under miR-708 treatment. In silico analysis of PC and NEPC tumor specimens suggested that the polycomb repressive complex subunit Enhancer of zeste homolog 2 (EZH2) was particularly overexpressed in NEPC. Notably, EZH2 bound to the miR-708 promoter and induced its silencing in NEPC. Inhibition of EZH2 prevented NE differentiation of PC cells. EZH2 expression was regulated by both Cyclin Dependent Kinase 1 (CDK1) and Wnt signaling. Silencing transcription factor 4 (TCF4), as a key protein in Wnt signaling, prevented NEPC formation. These results provide a molecular basis for the roles of miR-708 and EZH2 in NE differentiation in PC and highlight a new paradigm in NEPC formation and survival.

## Introduction

Prostate cancer (PC) is the second most commonly diagnosed and the fifth leading cause of cancer-related death in men^1^. Most patients with PC respond to androgen-ablation therapies, which exploit the androgen-sensitivity of PC cells by either lowering serum androgen levels or blocking androgen receptor (AR) activity, resulting in apoptotic cancer cell death. These treatments include gonadropin-releasing hormone analogs, which cause continuous stimulation of the pituitary gland leading to chemical castration with suppression of testosterone production, or anti-androgens, which directly block the AR. However, despite androgen deprivation, more than half of patients receiving androgen-ablation therapy (40% of patients with localized PC^2^) show tumor escape and progression to hormone-refractory, castration-resistant prostate cancer (CRPC), which is resistant to apoptosis and is largely untreatable^3–5^. Anti-androgen (enzalutamide and/or abiraterone) and taxane (docetaxel and/or cabazitaxel) -based chemotherapy is the only remaining therapeutic option for CRPC, with modest patient survival and palliative benefits^6^. It is therefore critical to understand the molecular mechanisms underlying the progression of PC to further the development of novel therapies to eradicate CRPC.

Tumor recurrence and progression into CRPC have been associated with significant enrichment of neuroendocrine (NE) cells within the bulk of the tumor, contributing to androgen-independent tumor progression^7^. NE differentiation is an oncogenic process leading to NEPC cells, which are epithelial-type prostate cells that share morphological and functional characteristics with neurons. They are unique in being non-proliferating, terminally differentiated, and AR-negative^8^. NEPC cells secrete neuronal markers, such as chromogranin A (CgA) and neuron-specific enolase (NSE)^9^. They also increase the proliferation of neighboring non-NE cancer cells in a paracrine manner, through the provision of hormonal peptide-mediated growth factors and anti-apoptotic properties^10^. It has been suggested that the expression of stem cell-associated markers, such as CD44 and Oct4, may support their roles in therapy evasion, tumor recurrence, and metastasis^11^. PC cells undergo NE differentiation as a result of several cell growth and microenivronmental conditions, including androgen-depletion, ionizing radiation, long-term chemotherapy exposure, adrenergic agents, and conditions activating the interleukin (IL)-6 signaling pathway^11^. Intermittent androgen-ablation therapy has signified the importance of NEPC cells in PC progression by reducing CgA serum levels through interfering with NE differentiation, and delaying the progression into advanced CRPC^12^. However, the mechanisms involved in NE cell resistance to apoptosis are not clearly understood, which presents a major impediment to the treatment of advanced CRPC. Thus, it is urgent to investigate the molecules or pathways inhibiting apoptosis in progressive PC to identify potential targets for the development of novel therapies to treat CRPC.

To this end, the current study investigated the mechanisms underlying the resistance of advanced CRPC to apoptosis. NE cells, which exhibit anti-apoptotic properties, are significantly enriched during PC progression, and we therefore used differentiated NEPC cells from PC cell lines, NEPC and prostate adenocarcinoma (PAC) tumor specimens, and a xenograft model to identify key molecules and pathways contributing to apoptosis resistance, with the aim of furthering the development of novel therapies to treat advanced PC.

Many miRNAs are deregulated in cancers, and they affect the expression of several genes including pro-apoptotic, anti-apoptotic, and tumor suppressor genes, and oncogenes, and have thus been proposed as key players in cancer etiology and progression^13^. We therefore also carried out miRNA expression profiling analysis of luminal PC cells and their corresponding NEPC cells to identify key miRNAs and other factors and signaling pathways involved in the resistance process.

## Results

### Conditioning with charcoal-stripped serum provides optimal conditions for induction of NE differentiation ex vivo

**Fig. 1:**
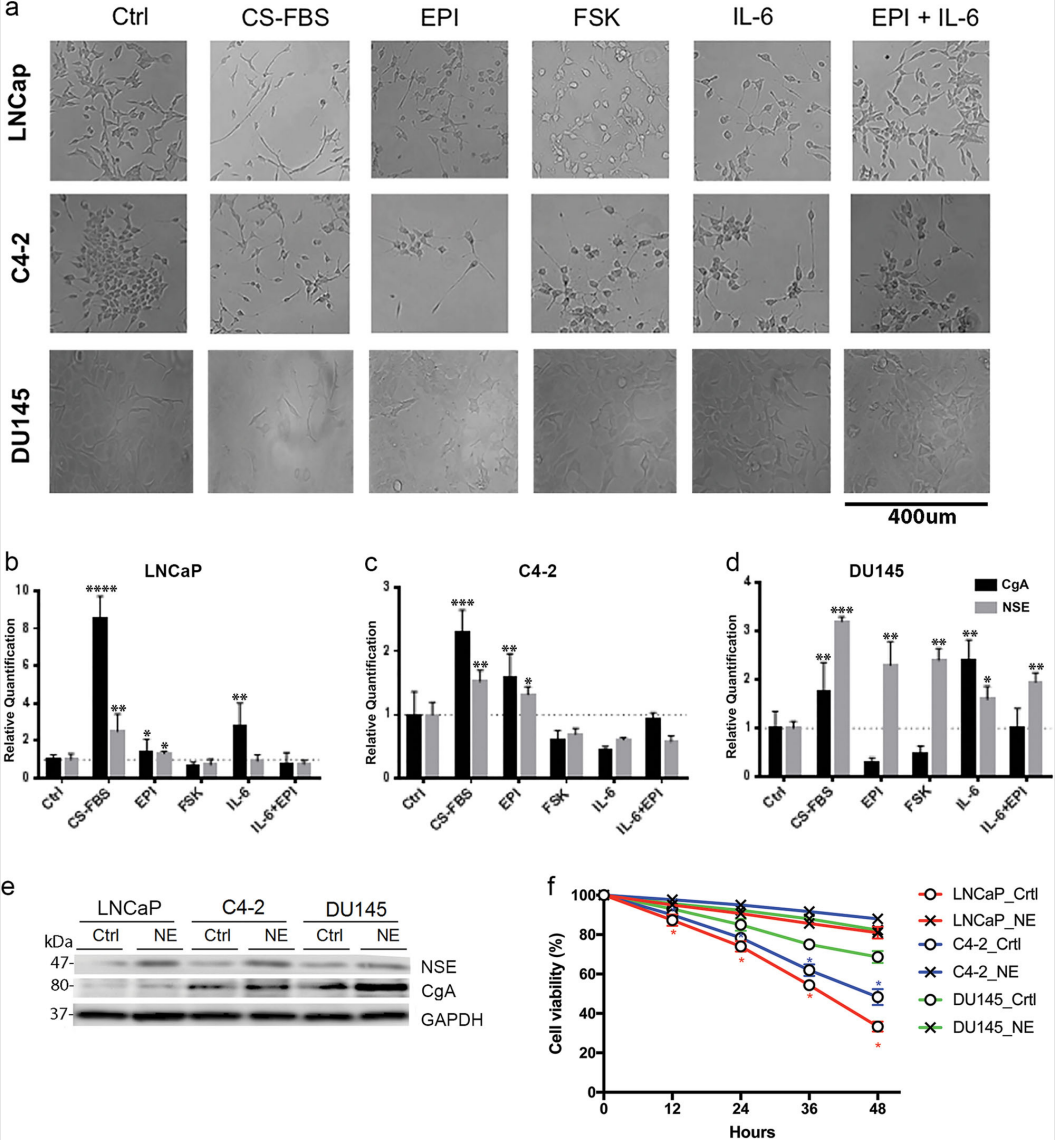
Optimal conditions for differentiation of PC cells to NEPC cells. PC cells were cultured in control medium (Crtl), phenol-red-free medium supplemented with 10% charcoal-stripped FBS (CS-FBS), or control medium supplemented with epinephrine (EPI), forskolin (FSK), IL-6, or both IL-6 and EPI. **a** Cells were imaged on day 3 following culturing in the respective media. Cells were cultured for 7 days in either control or NE-inducing medium, and expression of CgA and NSE were quantified by qPCR in **b** LNCaP, **c** C4–2, and **d** DU145 cells. *P < 0.05, **P < 0.01, ***P < 0.001, ****P < 0.0001 **e** Cells were cultured for 7 days in either control medium or phenol-red-free medium supplemented with 10% CS-FBS medium. NSE and CgA were analyzed by western blot. **f** MTT assays to detect viability of LNCaP, C4–2 and DU145 cells and their corresponding NEPC cells in the presence of 100 nM paclitaxel at 0, 12, 24, 36, and 48 h. *P < 0.05. Error bars represent the standard deviation of biological triplicates

LNCaP cells are androgen-dependent human PC cells derived from supraclavicular lymph node metastasis, C4–2 cells are androgen-independent aggressively metastatic PC cells isolated from bone^14^, and DU145 cells are human androgen-independent cells derived from brain metastasis^15^. Cells were cultured under conditions to promote NE differentiation, including androgen deprivation (phenol-red-free RPMI medium supplemented with 10% charcoal-stripped fetal bovine serum; CS-FBS), adrenergic agent epinephrine (EPI) or forskolin (FSK), IL-6, or their combination. Within 3 days of switching to androgen-deprived medium, cells gradually acquired an NE morphology with prolonged projections resembling cultured neurons (Fig. 1a). CS-FBS-treated cells expressed significantly higher levels of CgA and NSE compared with control cells and cells treated with IL-6, EPI, or FSK (Fig. 1b–d). Western blot confirmed that NEPC cells expressed higher levels of CgA and NSE compared with control cells (Fig. 1e). NEPC cells derived from LNCaP and C4–2 were resistant to the chemotherapeutic agent paclitaxel for up to 48 h compared to control PC cells (Fig. 1f). Phenol-red-free RPMI medium supplemented with 10% CS-FBS was the optimized condition to successfully induce NEPC from PC cells, and this condition was used in the following study for NEPC induction.

### miR-708 expression was significantly attenuated in NEPC cells

**Fig. 2:**
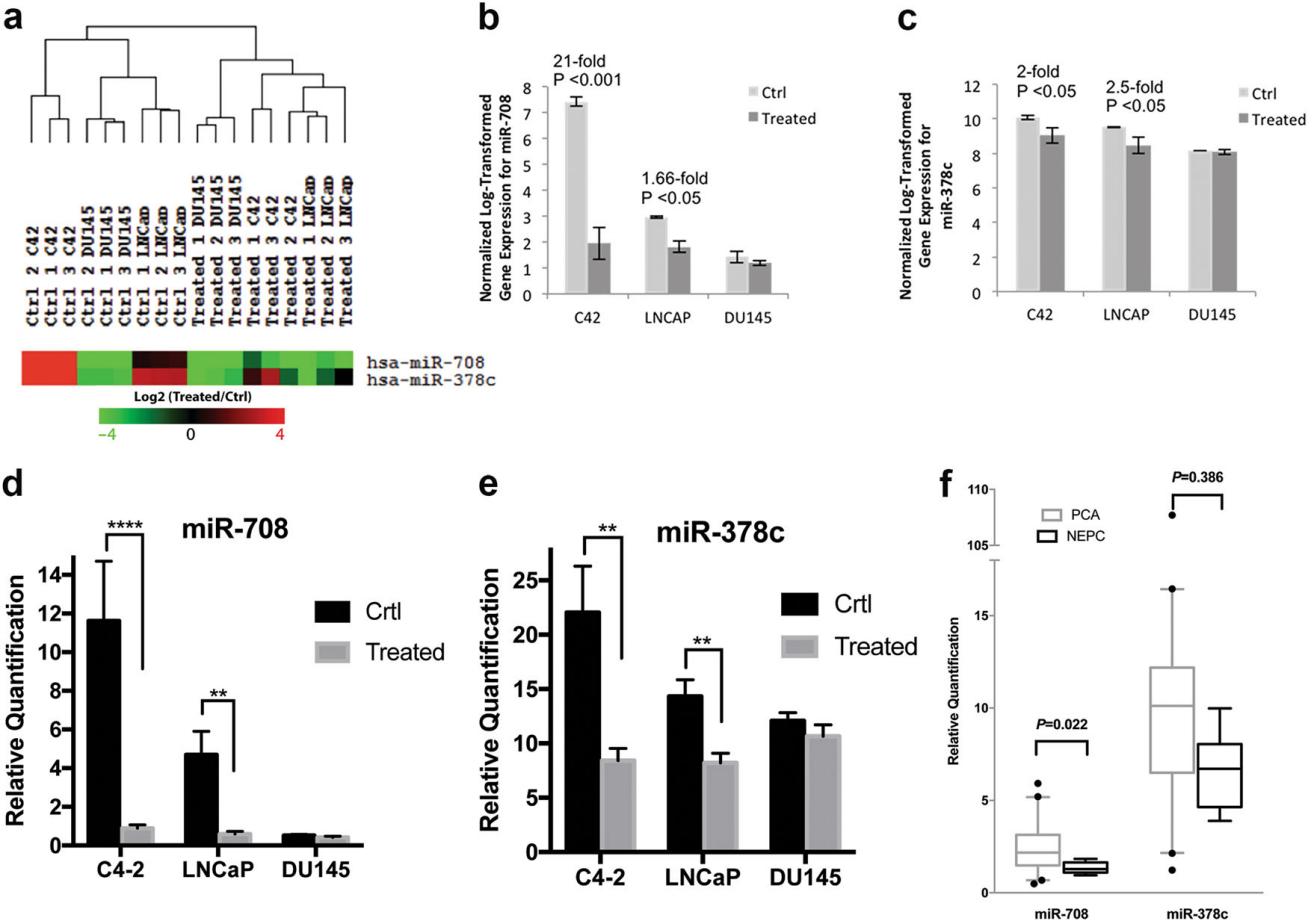
miRNA expression analysis. **a** Zoomed heatmap of miR-708 and miR-378c expression in control PC cells and charcoal-stripped FBS-treated (NE) cells for LNCaP, C4–2 and DU145. Fold increase and statistical difference in geometric mean intensities of expression of **b** miR-708 and **c** miR-378c in LNCaP, C4–2, and DU145 cells. qPCR analysis of **d** miR-708 and **e** miR-378c in control and CS-FBS-treated LNCaP, C4–2, and DU145 cells. **P < 0.01, ****P < 0.0001. **f** miRNA expression levels in 40 PAC and eight NEPC tumors were quantified by qPCR. Error bars represent the standard deviation of biological triplicates

Expression of pro-apoptotic (*Bax, Bak, Bid, Bik, Bim*, and *Puma*), anti-apoptotic (*Bclx, Bcl2, Mcl1, XIAP, CIAP2*, and *survivin*), and multi-drug resistance (*Foxo1, Abcg2, Abcb1* and *Mrp1*) genes were quantified by qPCR in NEPC and control cells (Supplementary Fig. S1), and no significant differences in the cumulative data were observed. NE differentiation is an adaptive phenotypic plasticity of cancer cells to develop resistance against apoptosis. miRNAs have shown great potential for regulating cancer cell plasticity^16,17^. We therefore investigated key miRNAs and their associated pathways responsible for this resistance by comparing the miRNA expression profiles of control LNCaP, C4–2, and DU145 cells with their corresponding NEPC cells. Two miRNAs, miR-708 and miR-378c, aroused our interest because both have been reported reduced expression in PC cells compared to normal prostate cells^18,19^ and showed further down-regulation in NEPC cells (Fig. 2a–c). qPCR showed that miR-708 was downregulated in CS-FBS-treated LNCaP (*P* < 0.01) and C4–2 (*P* < 0.0001) cells, respectively, but its expression was not altered in CS-FBS-treated DU145 cells (Fig. 2d). miR-378c was significantly downregulated in CS-FBS-treated LNCaP (*P* < 0.01) and C4–2 (*P* < 0.01) cells, with less fold changes compared with miR-708 assays, respectively, but not in DU145 cells (Fig. 2e). Notably, DU145 cells showed low expression of miR-708, similar to NEPC cells differentiated from LNCaP and C4–2 cells, possibly reflecting the fact that DU145 cells were established from a metastatic AR-negative CRPC, and thus share the same origin and characteristic as NEPC. We further validated the significance of the identified miRNAs in PC by qPCR in eight NEPC tissues and 40 PAC tissues. Expression levels of miR-708, but not miR-378c, were significantly reduced in NEPC tumors (Fig. 2f). All these results indicate that miR-708 might play an important role in shaping NEPC phenotype.

### miR-708 induced apoptosis in PC but not in all NEPC cells

**Fig. 3:**
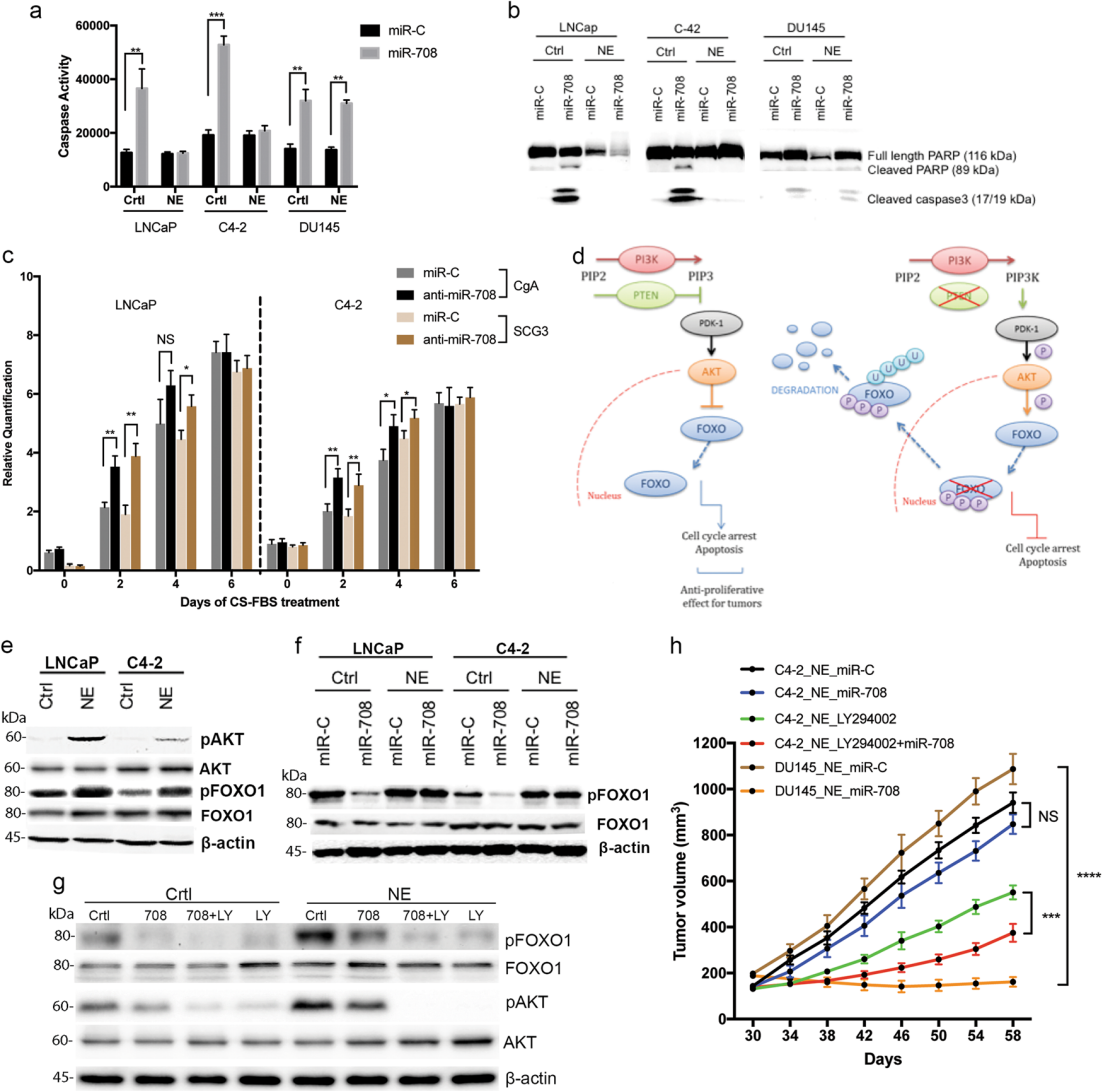
Effect of ectopic expression of miR-708 on apoptosis of PC cells and NEPC cells. **a** Caspase-3 activity assay 48 h after transfection. **P < 0.01, ***P < 0.001. **b** Western blotting analysis of full-length PARP, cleaved PARP, and cleaved caspase 3 after 48 h. **c** qPCR analysis of NEPC markers expression in cells transfected with miR-C or anti-miR-708 and then treated with CS-FBS for 2, 4, and 6 days. **d** PI3K/AKT pathway in the presence (left) or absence (right) of PTEN. *P < 0.05, **P < 0.01. (**e**) Western blotting analysis of phosphorylated AKT (pAKT) and phosphorylated FOXO1 (pFOXO1) levels in control and NEPC cells. **f** Western blotting analysis of FOXO1 and pFOXO1 levels in control PC and NEPC cells transfected with either control or miR-708 miRNA. **g** Control C4–2 and NEPC C4–2 cells were transfected with miR-708, treated with LY294002 (LY), or both. **h** DU145 or C4–2 cells were treated with CS-FBS for one week and then injected subcutaneously into nude mice to form solid, palpable tumors (day 30), and miR-708 mimic, control miR-C, LY294002 or the combination of miR-708 mimic and LY294002 were then delivered intratumorally every 4 days for 4 weeks. ***P < 0.001, **** *P* < 0.0001. Error bars represent the standard deviation of biological triplicates (**a**) or five replicates (**h**)

Downregulation of miR-708 was significantly associated with poor survival outcome and tumor progression in PC patients^18^. To assess the NEPC-specific role of miR-708, we first verified the role of miR-708 in the three PC cells used here. At 24 h after miR-708 transfection (miR-708 levels were determined in Supplementary Fig. S2a), all three cells showed apparent changes in cell morphology, with some cells becoming round and detached (Supplementary Fig. S2b). We determined if this change was related to apoptotic cell death by caspase-activity assay. miR-708-transfected three PC cells showed significantly increased caspase activity, indicating cell death by apoptosis, while in their corresponding NEPC cells, only DU145 derived ones showed change in caspase activity compared with cells transfected with control miR (miR-C) (Fig. 3a). This result was further validated by cleaved PARP and cleaved caspase 3 in miR-708-transfected LNCaP and C4–2 control cells and cleaved caspase 3 in miR-708-transfected DU145 control cells and their corresponding NEPC cells (Fig. 3b). Since miR-708 could induce apoptosis, we examined the role of miR-708 in NE differentiation by ectopic expression of anti-miR-708. The downregulation of miR-708 mediated by its inhibitor significantly promoted NE markers expression at early stage of differentiation (Fig. 3c). But with the progression of differentiation, the effect of anti-miR-708 become less significant and eventually disappeared (Fig. 3c). Next, we investigated the underlying mechanism how miR-708 induced apoptosis in PC cells but not in NEPC cells.

### miR-708 affected FOXO1 phosphorylation status

LNCaP and C4–2 do not express phosphatase and tension homolog (PTEN) tumor suppressor gene while DU145 does express PTEN, which plays a substantial role in prostate cancer. PTEN regulates the phosphatidylinositol 3-kinase (PI3K) pathway by blocking the phosphorylation of PIP3, resulting in the inhibition of AKT activity and the accumulation of unphosphorylated FOXO1, which promotes the transcription of cell cycle arrest and pro-apoptotic genes (Fig. 3d, left). In the absence of PTEN, the PI3K pathway becomes constitutively activated (Fig. 3d, right), leading to phosphorylation of FOXO1 (pFOXO1), which becomes sequestered in the cytoplasm and cannot direct the expression of pro-apoptotic genes. Given the importance of PTEN and FOXO1 in promoting and inhibiting cell death regulation, we investigated the role of miR-708 in this pathway. We first verified phosphorylation level of AKT and FOXO1 in PC cells and the corresponding NEPC cells. Phosphorylated AKT (pAKT) levels and phosphorylated FOXO1 (pFOXO1) level were increased following the induction of NE differentiation (Fig. 3e). We next examined the relationship between miR-708 and FOXO1. PC cells showed equivalent levels of FOXO1 with and without miR-708 overexpression, but pFOXO1 levels were significantly reduced (Fig. 3f). This was in agreement with the significant increase in cell death by apoptosis noted above (Fig. 3a, b), suggesting that overexpression of miR-708 attenuated the phosphorylation status of FOXO1. Ectopic expression of miR-708 had no discernable effect on pFOXO1 in NEPC cells (Fig. 3e). Given that AKT is over-activated in NEPC, we then determined the effect of simultaneously increasing miR-708 and inhibiting AKT on the phosphorylation of FOXO1. The combination of miR-708 and the AKT pathway inhibitor LY294002 almost completely suppressed FOXO1 phosphorylation in NEPC cells, while miR-708 alone only partially reduced its phosphorylation (Fig. 3g). We also examined the role of miR-708 *in vivo* by inoculating nude mice with NEPC cells derived from PTEN-efficient DU145 cells and PTEN-deficient C4–2 cells. After 4 weeks, the tumors were injected with miR-708 or miR-C every 4 days until 8 weeks. DU145 tumors stopped growing after miR-708 treatment, whereas C4–2 tumors continued to grow (Fig. 3h). However, miR-708 significantly slowed down the tumor growth when LY294002 was administrated simultaneously (Fig. 3h). These results suggested that NEPC cells were sensitive to miR-708 when AKT activity was inhibited.

### miR-708 reduced the frequency of CD44-expressing PC cells

miR-708 has been reported as a key negative regulator of a subpopulation of CD44-expressing PC cells (identified as cancer-initiating cells)^18^. However, this previous study did not consider the difference between PC and NEPC cells. We therefore investigated the impact of ectopic expression of miR-708 on the frequencies of CD44-positive cells among PC cells and corresponding NEPC cells. We assessed the frequency of CD44-positive cells by flow cytometry analysis 48 h after miR-708 transfection (Supplementary Fig. S3A). Ectopic expression of miR-708 reduced the frequency of CD44-positive cells and their corresponding NEPC cells (Supplementary Fig. S3B).

### miR-708 targeted Sestrin-3

**Fig. 4:**
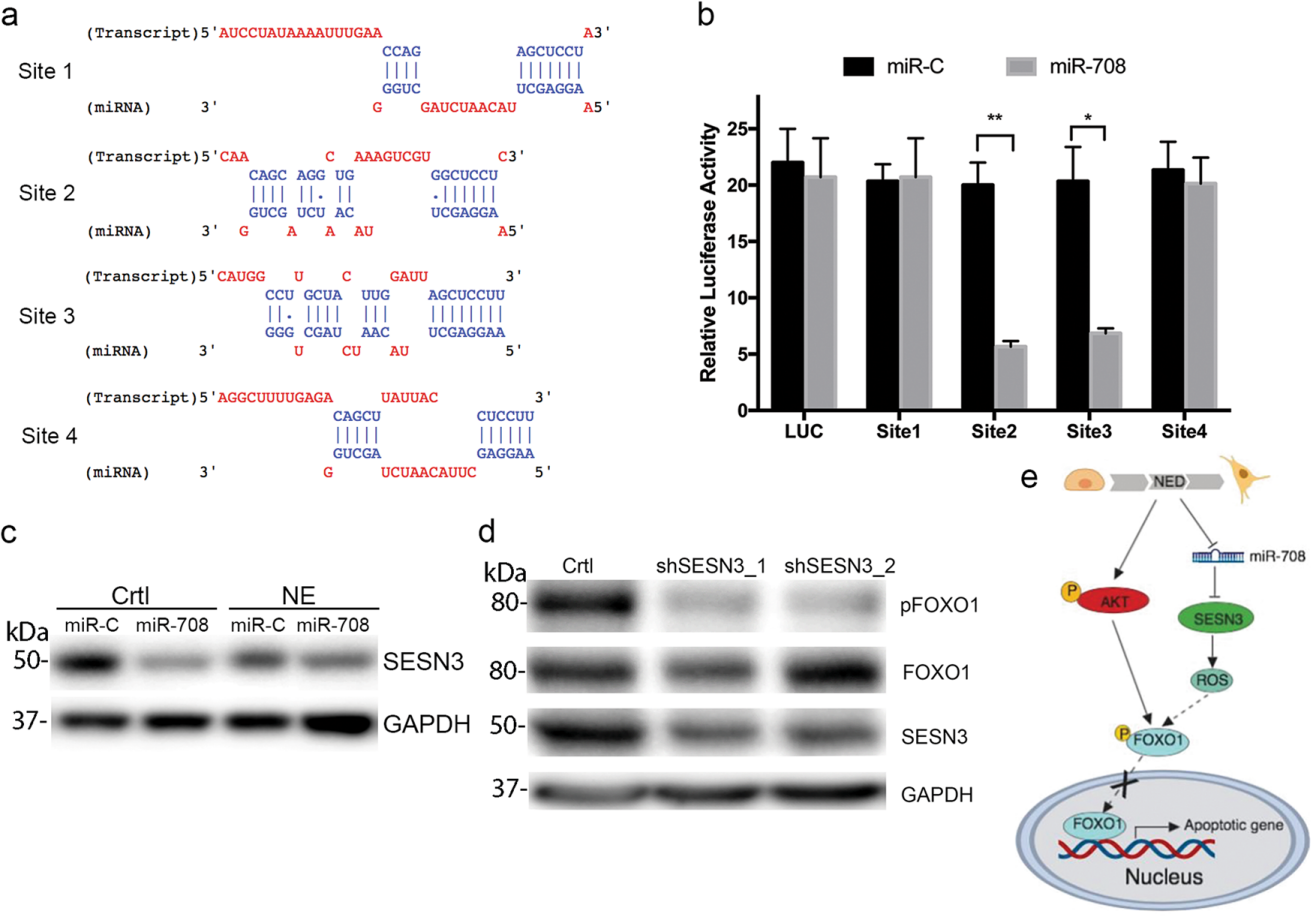
miR-708 targets *SESN3* in control PC and NEPC cells. **a** Schematic graph of 3′UTR of *SESN3* with putative binding sites of miR-708. The chromosome locations of sites 1–4 are chr11: 94903044–94903054, chr11: 94902521–94902545, chr11: 94899816–94899839, and chr11: 94903269–94903285, respectively. **b** Luciferase activity in HEK293T cells co-transfected with luciferase reporter plasmids containing four different predicted miR-708-binding sites on the *SESN3* 3′-UTR, respectively, together with miR-C or miR-708. Two-sided independent Student’s *t*-tests were used to compare expression difference between two groups. *P < 0.05, **P < 0.01. (**c**) Western blotting analysis of SESN3 in LNCaP control PC cells and NEPC cells transfected with miR-C or miR-708. **d** pFOXO1 levels in *SESN3*-silenced LNCaP cells. **e** Schematic diagram depicting effect of miR-708 on FOXO1 phosphorylation via SESN3 in control and NEPC cells. Error bars represent the standard deviation of biological triplicates

How miR-708 reduced FOXO1 phosphorylation was not clear. Therefore, we further examined the molecular mechanism of miR-708 in apoptosis by searching for potential targets of miR-708 using Tarbase v7.0^20^. Sestrin-3 (encoded by the *SESN3* gene), which ranks top in Tarbase as an experimentally identified target of miR-708, is associated with levels of intracellular reactive oxygen species (ROS). *SESN3* was shown to be elevated and to play an important role in CRPC^21,22^. To validate *SESN3* as a target gene of miR-708, we cloned four fragments containing the predicted miR-708-binding sites on the *SESN3* 3′ untranslated region (UTR) (Fig. 4a) into the psiCHECK vector 3′ of the luciferase reporter gene. The relative luciferase activities of site 2- and 3-, but not site 1- and 4-containing constructs were significantly suppressed by miR-708 (Fig. 4b). We also examined *SESN3* expression in control and NEPC cells transfected with miR-708. *SESN3* levels were decreased by miR-708 in both control and NEPC cells (Fig. 4c). *SESN3* and ROS have been shown to be reciprocally regulated^23^, and ROS also regulated FOXO1 activity^24^. To determine if *SESN3* affected FOXO1 activity, we silenced *SENS3* expression in LNCaP cells using a *SESN3*-specific short hairpin RNA (shRNA) (Supplementary Fig. S4A) and showed that FOXO1 phosphorylation was compromised in *SESN3*-silenced cells (Fig. 4d). These results suggested that miR-708 could potentially inhibit FOXO1 phosphorylation and then activate FOXO1-induced apoptosis by reducing SESN3, while NE differentiation of PC cells could influence FOXO1 activity through both miR-708/SESN3 and AKT pathways (Fig. 4e).

### EZH2 targeted miR-708 in NEPC and was essential for NEPC formation

**Fig. 5:**
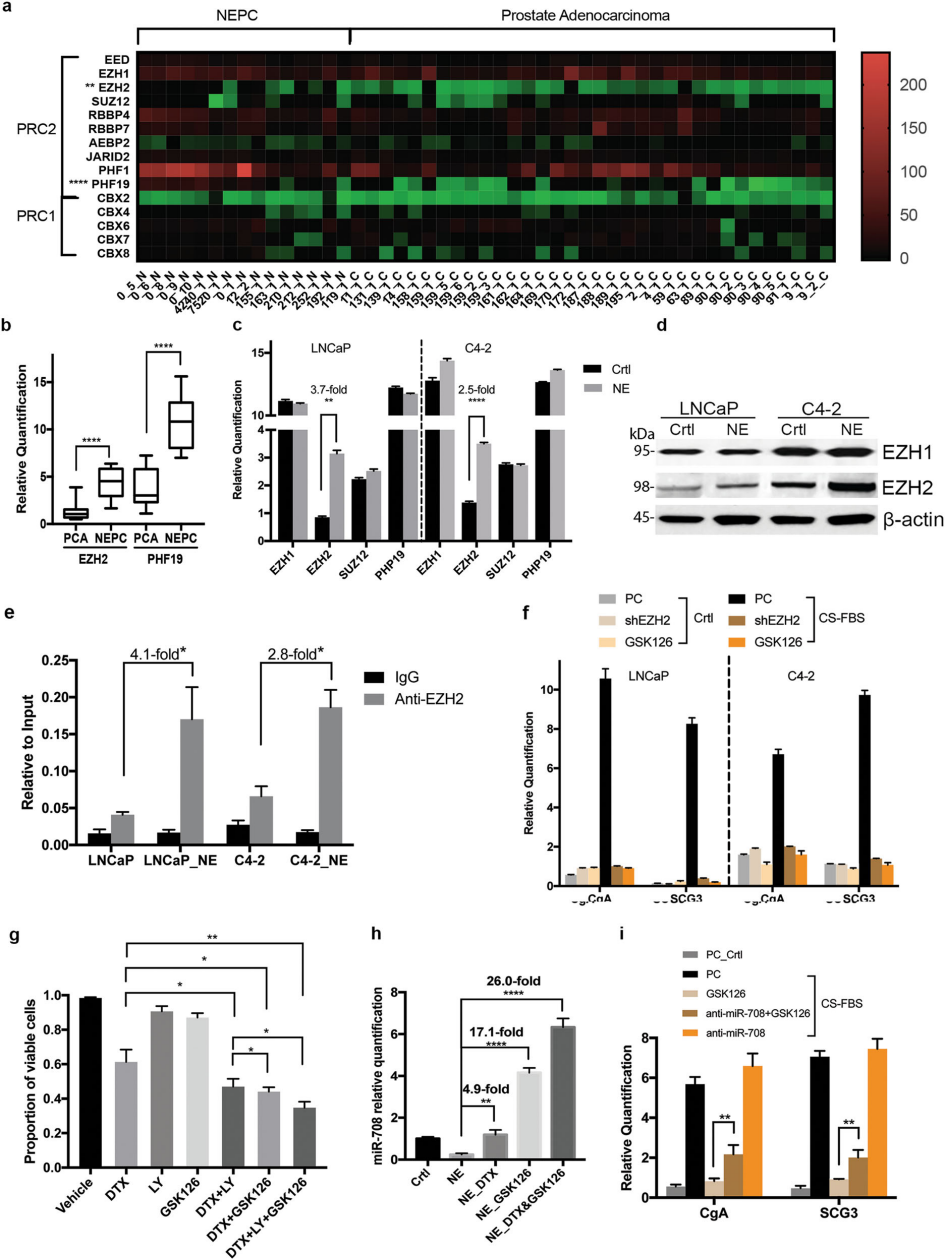
EZH2 is required for NEPC cells. **a** Expression profiles of PRC subunits in PAC and NEPC samples. *P*-value adjusted by Bonferroni method. **P < 0.01, ****P < 0.0001. (All values below 0 were close to 0 and the green color was therefore not distinguishable on the right bar). **b** qPCR analysis of EZH2 and PHF19 expression in our tumor specimen cohort including 40 PAC and eight NEPC samples. ****P < 0.0001. **c** qPCR analysis of expression of four PRC genes in control PC and NEPC cells. **P < 0.01, ****P < 0.0001. **d** Western blotting of EZH1 and EZH2 in control PC and NEPC cells. **e** ChIP enrichment analysis of EZH2-binding site on miR-708 in control PC cells and NEPC cells. **f** Effect of EZH2 knockdown and EZH2 inhibition by GSK-126 on NEPC markers expression. EZH2-knockdown PC cells were treated with NEPC-inducing medium for 6 days, or control cells were treated with NEPC-inducing medium with GSK126 (2.5 μM) for 6 days. NEPC phenotype was evaluated by qPCR of NEPC markers CgA and SCG3. **g** Effects of EZH2 inhibitor GSK126, docetaxel (DTX), PI3K/AKT pathway inhibitor LY294002 alone, combination of GSK126 with DTX or LY294002, or DTX + LY294002 on cell viability of NEPC cells derived from C4–2. NEPC cells were treated with vehicle, GSK126 (5 μM), DTX (10 nM), LY294002 (LY, 5 μM), DTX + LY294002, DTX + GSK126, or DTX + GSK126 + LY for 48 h, and the percentages of viable cells were counted and calculated. *P < 0.05, **P < 0.01. **h** miR-708 expression in NEPC cells derived from C4–2 treated with the indicated drugs for 24 h. **P < 0.01, ****P < 0.0001. i Effects of anti-miR-708 on NEPC markers expression when EZH2 was inhibited. C4–2 cells were transfected with anti-miR-708 and then treated with NEPC-inducing medium for 6 days with or without GSK126. **P < 0.01. Error bars represent the standard deviation of biological triplicates

Although miR-708 was significantly and specifically reduced in NEPC cells, ectopic expression of miR-708 in these cells had no substantial effect. We therefore investigated the mechanism through which miR-708 was downregulated in NEPC cells, to help shed light on the mechanisms underlying NEPC formation. On the UCSC genome browser, we identified an upstream region of miR-708 that showed strong binding to EZH2 (Supplementary Fig. S5), the core subunit of the polycomb repressor complex (PRC). PRC was previously suggested to regulate the expression of miR-708, and miR-708 levels were reduced by overexpression of the PRC subunit SUZ12 in breast cancer^25^. We therefore analyzed the gene expression levels of subunits of the PRC in PAC and NEPC tumors^26^ to determine if PRC subunits other than EZH2 were involved in the regulation of miR-708 in NEPC. In silico expression analysis of PRC1 and PRC2 subunits in PAC and NEPC^24^ suggested that EZH2 and PHF19 were significantly upregulated in NEPC (Fig. 5a). We validated the expression levels of these two genes in our own specimen cohort and found similar results to that of the in silico analysis (Fig. 5b) However, only EZH2 was upregulated in the induced NEPC cells (Fig. 5c, d). Chromatin immunoprecipitation (ChIP) analysis showed that EZH2 strongly bound to the miR-708 promoter in NEPC cells but weakly in control cells (Fig. 5e). C4–2 and LNCaP cells were infected by lentiviral shRNAs against EZH2 to establish EZH2-silenced PC cell lines (Supplementary Fig. S4B). However, miR-708 was not upregulated by EZH2 silencing in PC cells (data not shown).

To determine if miR-708 was upregulated after EZH2 silencing in NEPC cells, we examined NE markers in EZH2-silenced cells in NEPC-inducing media. However, the expression of NE markers, CgA and SCG3, was not induced by EZH2 silencing (Fig. 5f). Similarly, the expression of NE markers was not induced when cells were treated with the EZH2 inhibitor GSK126 (Fig. 5f). These data suggested that EZH2 might be essential for the differentiation of NEPC cells from PC cells. We also determined the effect of the EZH2 inhibitor on NEPC cell viability and showed that the inhibitor had only a minimal effect (Fig. 5g). However, the EZH2 inhibitor significantly reduced the proportion of viable cells with the addition of PC chemotherapy agent docetaxel and/or PI3K/AKT inhibitor LY294002 (Fig. 5g). We then quantified the effects of drug treatment on miR-708 expression in NEPC cells. Docetaxel treatment significantly induced miR-708 expression and killed NEPC cells, while GSK126 substantially induced miR-708 expression, despite minimal toxicity to NEPC. The combination of docetaxel and GSK126 induced a > 25-fold increase in miR-708 expression with synergistic toxicity to NEPC cells (Fig. 5h). These results also supported the role of miR-708 in apoptosis. miR-708 inhibitor partially restored the NE marker expression of EZH2 inhibition caused by GSK126 (Fig. 5i), indicating a role of miR-708 in NE differentiation.

### Inactivated CDK1 signaling and activated WNT signaling drive EZH2 expression in NEPC

**Fig. 6:**
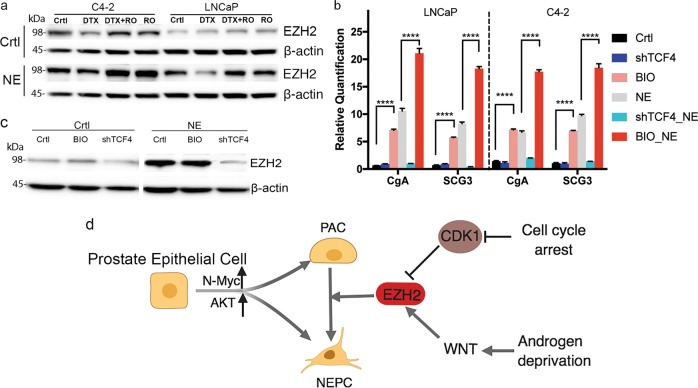
Effects of CDK1 and Wnt signaling on EZH2 expression in NEPC cells. **a** Effect of CDK1 activation on EZH2 expression in control and NEPC cells. C4–2 and LNCaP cells and their corresponding NEPC cells were treated with docetaxel (DTX; 10 nM) for 24 h followed with or without the CDK1 inhibitor RO3306 (RO) 24 h. The cells were then subjected to western blotting analysis. **b** Effects of WNT signaling activation and deactivation on NEPC markers expression. C4–2 and LNCaP cells were treated with GSK-3 inhibitor IX (BIO, 2.5 μM) for 1 day following 1 day culture in control or NEPC-inducing medium. TCF4-knockdown C4–2 and LNCaP cells were cultured in NEPC-inducing medium for 6 days. The cells were then lysed for qPCR analysis. ****P < 0.0001. **c** Western blotting analysis of LNCaP control and NEPC-induced cells. **d** Schematic diagram depicting the upstream and downstream signaling of NEPC. Error bars represent the standard deviation of biological triplicates

Given that EZH2 was shown to be essential for NE differentiation of PC cells, we investigated why it was induced during NE differentiation. Androgen depletion and contact inhibition, which induce NE differentiation, are associated with cell cycle arrest^27,28^. Deregulation of the cell cycle by inhibiting CDK1 promoted NE differentiation^29^. We therefore determined if CDK1 signaling affected EZH2 levels in this process. We activated CDK1 using the CDK1 regulator, docetaxel^29^, because it is a commonly used agent in PC treatment. EZH2 was degraded upon docetaxel treatment and restored by the CDK1 inhibitor RO3306 (Fig. 6a) in both control and NE cells. In addition to CDK1 signaling, Wnt signaling is also frequently deregulated in NEPC^30^. The Wnt signaling agonist, GSK-3 inhibitor IX (BIO), significantly induced the expression of the NE markers, CgA and SCG3, in PC cells and their corresponding NEPC cells (Fig. 6b) compared with cells without BIO treatment. BIO also upregulated EZH2 levels in control PC cells (Fig. 6c). Inhibition of GSK-3 could lead to the accumulation of its substrate, TCF4. To confirm the role of Wnt signaling in NEPC, we therefore examined the role of TCF4 in NEPC differentiation and its relationship with EZH2. TCF4 expression was silenced by shRNA against TCF4 in LNCaP and C4–2 cells (Supplementary Fig. S4C), and the TCF4-knockdown cells were then subjected to NE differentiation induction. Quantitative analysis of NE markers suggested that the NE phenotype was not induced in these cells by TCF4-knockdown (Fig. 6b), probably because EZH2 expression was inhibited by TCF4 silencing (Fig. 6c). Overall, these findings suggest that EZH2 plays a pivotal role during NEPC formation (Fig. 6d).

## Discussion

**Fig. 7:**
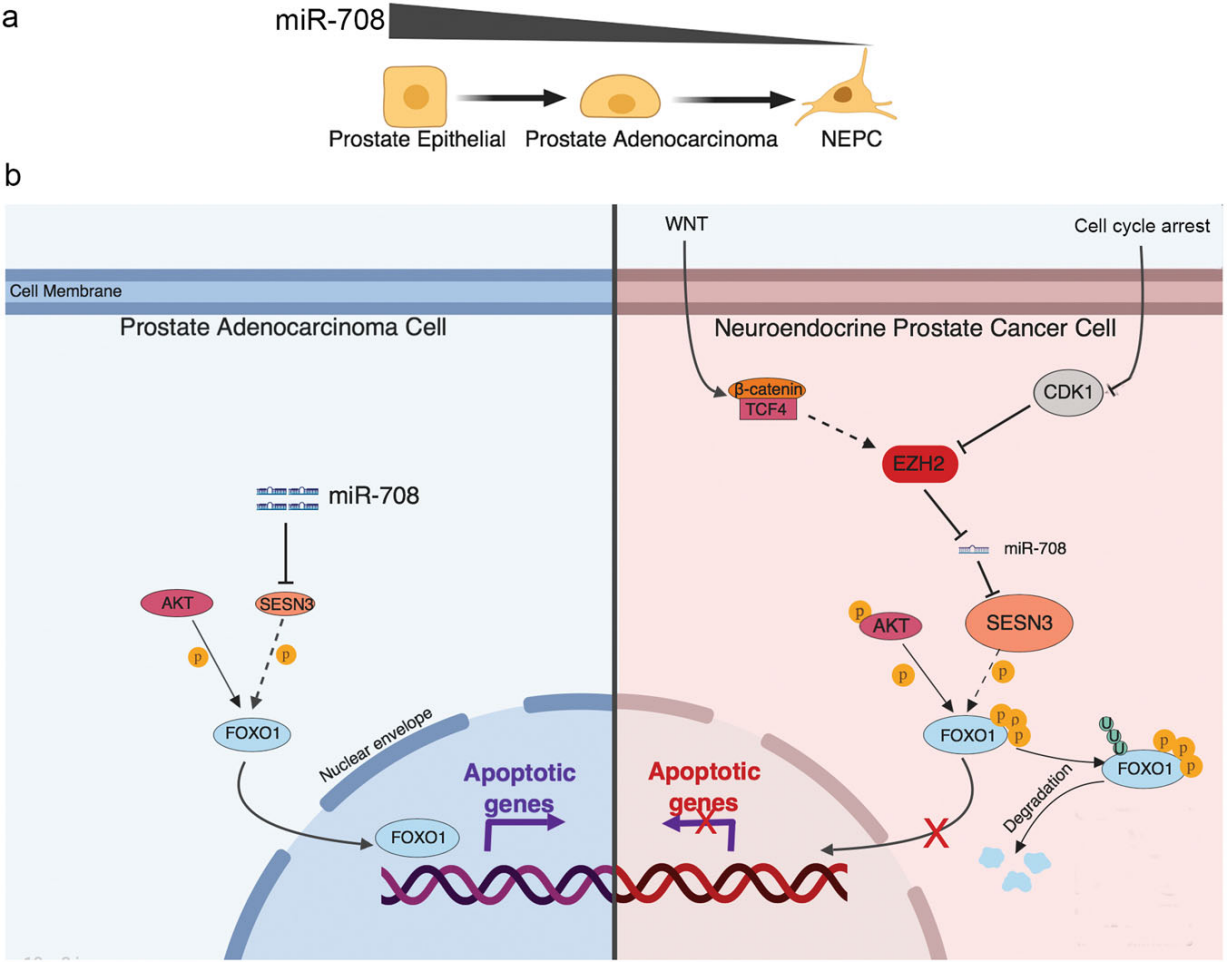
**a** miR-708 expression gradient is reduced during the transformation from normal prostate epithelial cells through adenocarcinoma cells to NEPC. **b** Schematic diagram depicting miR-708 and its associated upstream and downstream signaling in PAC cells and NEPC cells

Clinical evidence suggests that treatment-based AR suppression may lead to the development of highly lethal NEPC tumors in a subset of patients with CRPC^31^. Although several rigorous studies have shown that N-MYC is a driver gene for NEPC^32–34^, N-MYC amplification only occurred in 40% of NEPC tumors^32^, and was therefore incapable of explaining the full spectrum of NEPC. We therefore performed the current multifaceted study to identify potential candidate markers for NEPC and reveal the mechanism underlying NEPC formation. Our studies highlight the roles of miR-708 and EZH2 as key pathways in NE differentiation, potentially indicating progression toward an NE phenotype in patients with PC. EZH2 may thus be a potentially strong therapeutic target for the treatment and/or prevention of NEPC. The major findings are summarized in Fig. 7a,b.

Although the roles of miRNAs in PC have been well studied, limited information is available on the role of miRNAs in NEPC. miR-708 has been identified as an anti-tumor miRNA inducing apoptosis in PAC cells^18^. We revealed that reduced miR-708 expression was a consequence of NE differentiation. miR-708 was also downregulated in CRPC stem cells^35^. Hormone-depleted medium induced NE differentiation and increased the incidence of NEPC in metastatic CRPC^36^, suggesting that the appearance of NEPC could be a resistance mechanism to AR-targeted therapy. NEPC has a rare morphological variant termed small cell neuroendocrine carcinoma (SCNC)^36^, which shares a common transcriptional program with prostate stem cells^37^. Taken together, these data indicate that loss of miR-708 expression could be used to monitor the development and progression of PC in high-risk populations and PC patients. Furthermore, therapeutic strategies targeting cancer stem cells have great potential for treating advanced PC.

As epigenetic regulators, EZH2 and other PRC2 components are vital for maintaining stemness. The similarities in expression profiles between NEPC and prostate stem cells indicate that EZH2 might also be important for NEPC differentiation. EZH2 was overexpressed in NEPC mouse models, and was associated with an NEPC transcriptional program^32^. The current results showed that EZH2, but not other PRC subunits, was upregulated when PC cells transdifferentiated into NEPC cells, strongly indicating a unique role for EZH2 in NEPC. We also showed that EZH2 was not only important for maintaining the NEPC phenotype, but was also required for the formation of NEPC originating from PCA. A recent report demonstrated that the combined loss of function of *P53* and *RB1* drove lineage plasticity in PC cells and androgen therapy resistance, and EZH2 was indicated as a shared downstream molecule in these key events^38^. *P53* and *RB1* loss are common in NEPC^30,32^. By searching the upstream events of EZH2, we showed that CDK1/Rb1^39^ and Wnt/TCF4 could potentially regulate EZH2 levels. Notably, docetaxel could activate CDK1 and further lead to the degradation of EZH2, suggesting its additional benefit as a treatment for reducing the possibility of NEPC in PC. TCF4, as a key component of Wnt signaling, was overexpressed in CD49f^Hi^ SCNC cells^34^ and NEPC cells^25^, indicating a role for the Wnt signaling in NEPC formation. We confirmed this role in NEPC by activating or abrogating Wnt signaling in PC cells, resulting in increased expression of NEPC markers and inhibition of transformation of NEPC, respectively. Coincidently, in-depth genomic and transcriptomic analyses of metastatic CRPCs revealed that alterations in Wnt signaling occurred in 18% of patients^40^, which is similar to the percentage of NEPC in CRPCs.

EZH2 appears to be a common factor in the signaling pathways triggered by all the key molecular events in NEPC, including N-MYC amplification, cell cycle arrest, activated Wnt signaling, and loss of *P53* or *RB1*. Targeting EZH2 could thus help in the management of CRPC that gives rise to NEPC. Furthermore, we showed a beneficial response to the addition of an EZH2 inhibitor to NEPC treatment, although the EZH2 inhibitor alone had little effect. A recent report suggested that EZH2 was involved AR signaling^41^, which might explain the combinational effect.

In conclusion, our study revealed the importance of epigenetic regulation in NEPC formation and provided viable strategies for the therapeutic management of PC and NEPC.

## Materials and methods

### Study approval

Forty PAC and eight NEPC tumor RNA samples were obtained from the WCMC-Qatar Genetic Medicine Database and the Arab Breast and Prostate Cancer Consortium Bio-Repository. All protocols were approved by the Weill Cornell Medicine-Qatar Institutional Review Board (Doha, Qatar). All subjects signed informed consent documents for participation in the study.

### Cell lines and reagents

The C4–2 cell line was a gift from Leland Chung (Emory University). All other cell lines were obtained from the American Type Culture Collection (ATCC). All antibodies were purchased from Cell Signaling Technology (CST), unless otherwise specified. Enolase-2 (NSE) rabbit antibody (CST, 9536), CHGA (CgA) rabbit antibody (CST, 60893), PARP rabbit antibody (CST, 9542), Caspase-3 (D3R6Y) rabbit antibody (CST, 14220), phosphor-AKT (S473) (EP2109Y) rabbit antibody (Abcam, ab81283), AKT rabbit antibody (CST, 9272), phospho-FOXO1(Ser256) (E1F7T) rabbit antibody (CST, 84192), FOXO1(C29H4) rabbit antibody (CST, 2880), SESN3 rabbit antibody (ABclonal, A5164), EZH1 (D7D5D) rabbit antibody (CST, 42088), EZH2 (D2C9) Rabbit antibody (CST, 5246), β-Actin rabbit antibody (CST, 4967), The dilution of the primary antibody was 1:1500. LY294002, GSK126, GSK-3 inhibitor IX, and IL-6 were from Merck Millipore. Docetaxel was from Selleckchem. All other chemicals and reagents were purchased from Sigma.

### Cell culture

All cell lines were maintained in RPMI-1640 medium with 10% FBS at 37 °C in a humidified incubator with 5% CO_2_. NE phenotype was induced in RPMI-1640 medium without phenol red with 10% charcoal-stripped FBS for 7 days, and the medium was changed every 2 days.

### Cell proliferation and apoptosis assays

Cell proliferation was quantified by measuring viable cells by MTT assay. The cells were incubated in growth medium with 1 mg/mL MTT for 3 h at 37 °C. The medium was then replaced with 100 μL dimethylsulfoxide. The plate was covered with tinfoil and agitated on an orbital shaker for 15 min. Absorbance was recorded at 570 nm with a filter reference at 620 nm using an EnVision Multilabel Plate Reader (PerkinElmer).

Treated cells were lysed in lysis buffer and apoptosis was assessed by measuring caspase-3 activity using the fluorogenic substrate Ac-DEVD-AFC as described previously^42^. Briefly, cells were collected and lysed in caspase lysis buffer (1% Nonidet P-40, 150 mM NaCl, 20 mM HEPES, 1 mM EDTA, 1 mM dithiothreitol, 5 μg/mL aprotinin, leupeptin, and pepstatin). Fluorescence was recorded every 15 min for 1 h, and caspase activity was expressed in arbitrary units. Presented results were confirmed by at least two independent experiments.

### Microarray analysis

Total RNA was extracted from cells using TRIzol (Invitrogen). The purity and concentration of the total RNAs were qualified using a Bioanalyzer 2100 (Agilent). Total RNA was fluorescence-labeled using an Affymetrix FlashTag Biotin RNA labeling kit, hybridized using an Affymetrix GeneChip™ miRNA 3.0 Array overnight, and then washed and stained using an Affymetrix GeneChip Hybridization Wash and Stain Kit following the manufacturer’s protocol. The hybridization results were scanned and images were assessed for quality using an Affymetrix GeneChip Scanner. Cell file data from each microarray were exported, normalized using Robust Multi-array, and further analyzed using BRB-Array Tools^43^.

### Flow cytometry

Cells were washed in staining solution containing DPBS (Ca^2+^- and Mg^2+^-free) with 1 mM EDTA, 25 mM HEPES, and 0.5% FBS, and incubated with CD44-PE antibodies (BD Biosciences) for 30 min at 4 °C. Cells were rinsed twice in staining solution and analyzed using an LSR Fortessa cell analyzer (BD Biosciences).

### qPCR and western blot

Total RNA was isolated using TRIzol regent (Invitrogen) according to the manufacturer’s instructions. For mRNA expression, total RNA was reverse transcribed into cDNA using an oligo 18 T primer, and gene expression levels were then measured by qPCR with a GoTaq^®^ 2-Step RT-qPCR System for SYBR Green-based detection. The *HPRT1* gene was used as a reference. The primer sequences are listed in Table EV1. miRNA expression levels were quantified by miRCURY LNA miRNA PCR Assays (Qiagen) using U6 as a reference, according to the manufacturer’s instructions. All qPCR assays were performed using an Applied Biosystems^®^ QuantStudio 6 Flex Real-Time PCR System.

Western blotting was performed using standard protocols. Briefly, cells were lysed with RIPA buffer and prepared in 1× sodium dodecyl sulfate (SDS) sample loading buffer, boiled for 10 min at 95 °C, separated on a 10% SDS polyacrylamide gel, and transferred to a polyvinylidene difluoride membrane. Membranes were blocked with 5% milk in TBST for 1 h at room temperature, incubated in primary antibody diluted in TBST overnight at 4 °C, washed three times for 5 min each with TBST, and incubated for 1 h with secondary antibody. Membranes were washed three times for 10 min each with TBST and the chemiluminescence signal was detected by Immobilon^®^ Crescendo (Millipore) and imaged by ChemiDOC™ MP (Bio-Rad).

### miRNA mimics and shRNA

Oligonucleotides for Ambion^®^ hsa-miR-708–5p mimics (miR-708), negative control (miR-C), and hsa-miR-708 inhibitors (anti-miR-708) were transfected into cells using TransIT-X2^®^ transfection reagent (Mirus) at a final concentration of 25 nM, following the manufacturer’s protocol. shRNA constructs were established using pLKO.1 vector (gift from David Root) (Addgene plasmid #10878; http://n2t.net/addgene:10878; RRID: Addgene_10878). The targeting sequences are listed in Supplementary Table 1. PC cell lines carrying the shRNA constructs were established following the Addgene pLKO.1 vector protocol (http://www.addgene.org/tools/protocols/plko) using 1 μg/mL puromycin as the selection drug.

### ChIP

ChIP was performed with anti-EZH2 antibody using a SimpleChIP kit (Cell Signaling) following the manufacturer’s protocol. Briefly, cells were fixed with formaldehyde to cross-link proteins to DNA. Cells were lysed and chromatin was sonicated briefly, followed by digestion with micrococcal nucleases into 150–900 bp DNA/protein fragments. Anti-EZH2 antibodies were added and the complex was co-precipitated and captured by Protein G Agarose. Cross-links were reversed and DNA was purified. The ChIP products were then analyzed by qPCR. The ChIP-qPCR primer sequences are listed in Supplementary Table 1.

### Luciferase activity assay

Three different *SESN3* 3′-UTR fragments containing the putative miR-708-binding sites were synthesized and cloned into the psiCHECK luciferase vector (Promega) containing firefly luciferase. Cells were seeded into 24-well plates 24 h before transfection. A total of 500 ng of luciferase reporter plasmid were transfected into each well together with 20 ng of pRL-TK vector (Promega) and 60 pmol of oligonucleotides. Cells were harvested 48 h later and firefly and *Renilla* luciferase activities were measured by dual-luciferase reporter assay (Promega) on CLARIOstar (BMG Labtech).

### Tumor xenograft model

Five castrated nude mice (4-week-old; Institute of Zoology, Chinese Academy of Sciences) for each group received subcutaneous injections of 1 × 10^7^ CS-FBS-treated DU145 or 5 × 10^6^ CS-FBS-treated C4–2 cells, mixed with 1× Matrigel (BD Bioscience) in a volume of 100 μL, in both lateral flanks. Once palpable tumors developed, tumor width and length were measured twice a week using calipers. When the tumors reached an average volume of 150 mm^3^, 6.25 mg Ambion^®^ miRNA mimics (miR-708/miR-C) complexed with 1.6 mL siPORT Amine transfection reagent (Ambion) or 50 mg/kG LY294002 in 50 μL phosphate-buffered saline was delivered intratumorally at 4-day intervals. The dosage was selected based on previous results^18^. Mice were euthanized 2 days after the last treatment (day 58).

### Statistics

Expression differences in miRNAs and other genes between PCA and NEPC tumors or between control PC cells and NEPC cells were analyzed using independent Student’s *t*-tests. Time course difference between two groups were analyzed using two-way Anova analysis. All statistical tests were two-sided. A *P*-value less than 0.05 is considered significant. Statistical analyses were performed using GraphPad Prism 7. Unless otherwise stated, error bars represent the standard deviation of biological triplicates.

## Supplementary information

Supplemental Materials
