# Supplementary material for: Targeting Wnt/EZH2/microRNA-708 signaling pathway inhibits neuroendocrine differentiation in prostate cancer
Source: Cell Death Discov. 2019 Sep 30;5:139. doi: 10.1038/s41420-019-0218-y (PMC6768854; doi:10.1038/s41420-019-0218-y)
Supplement: Supplementary file 1 — Supplemental Materials [file 41420_2019_218_MOESM1_ESM.docx]

**Supplementary Data**

**Supplementary Figures**


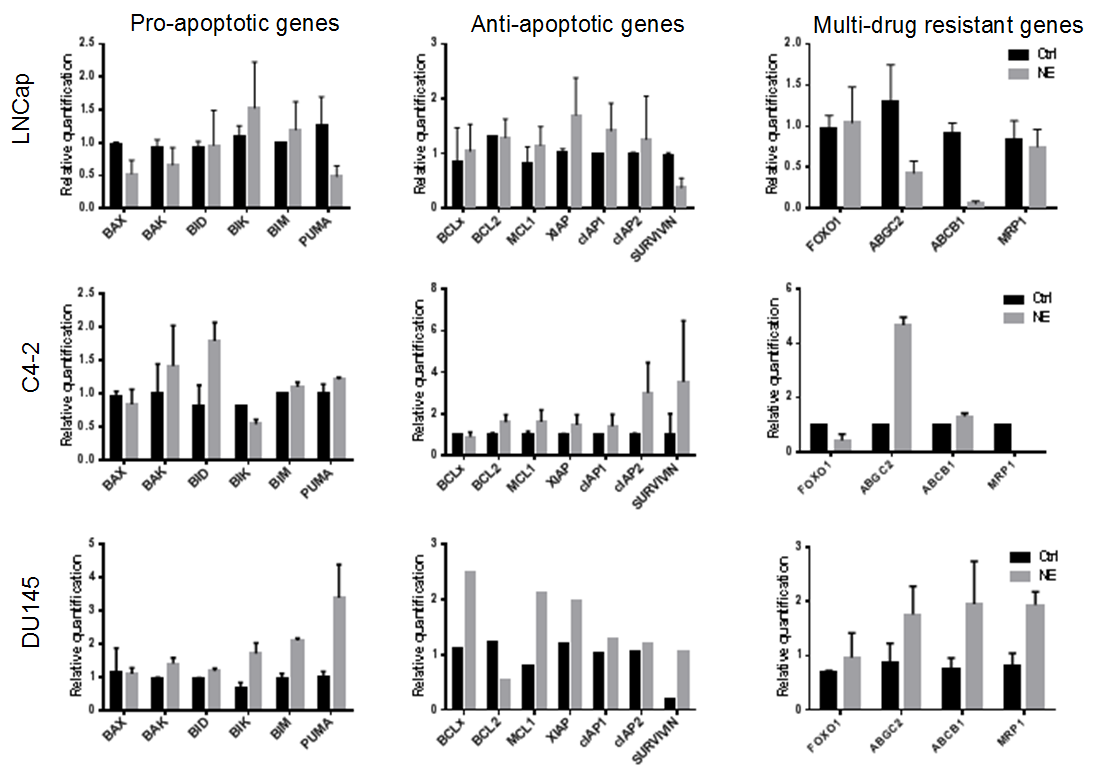


**Supplementary Figure 1**. Cells were cultured for 7 days in either control medium or phenol-red-free medium supplemented with 10% CS-FBS medium. Gene expression profiling of apoptosis-related genes in control PC and NEPC cells by qPCR.

a


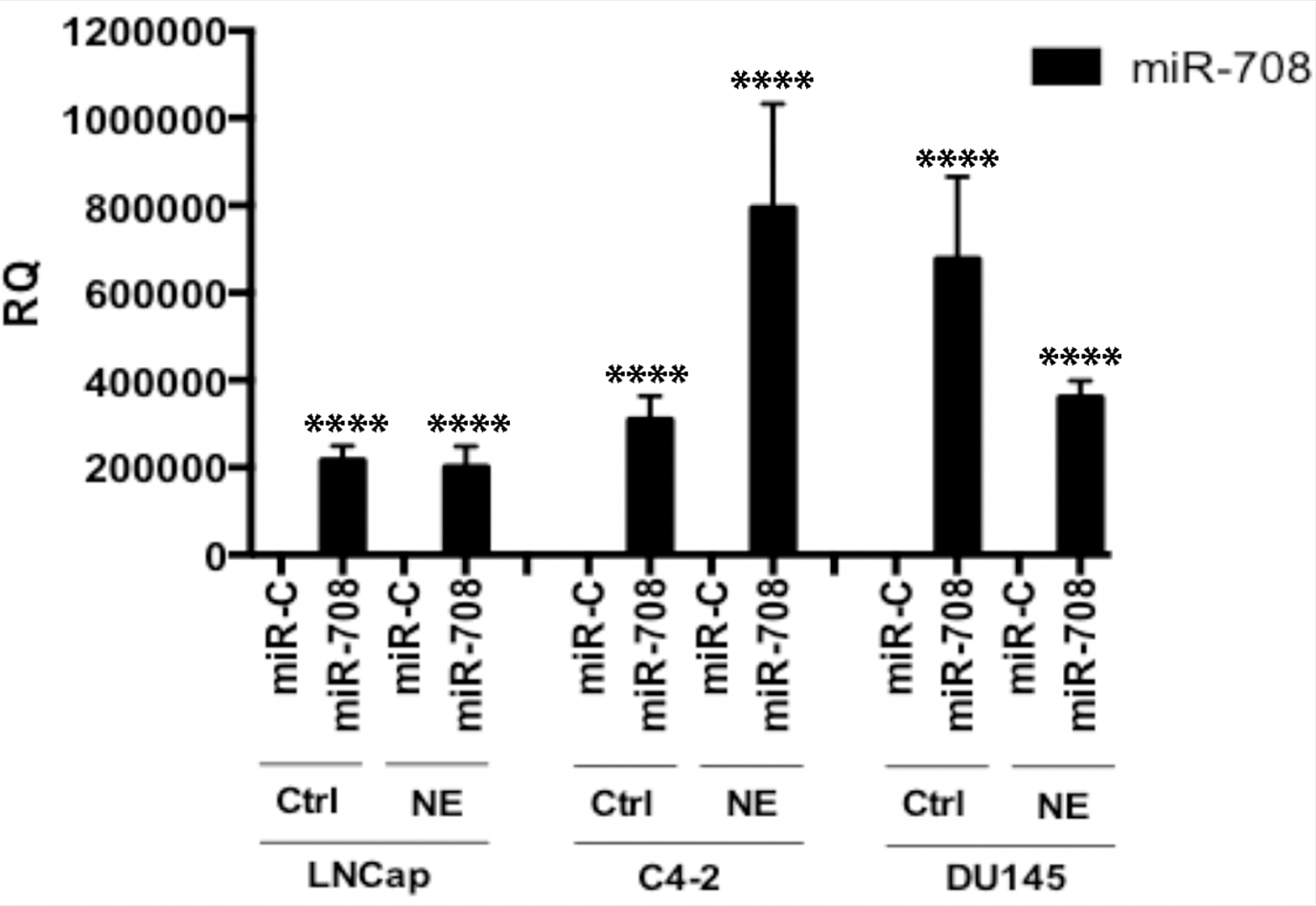


b


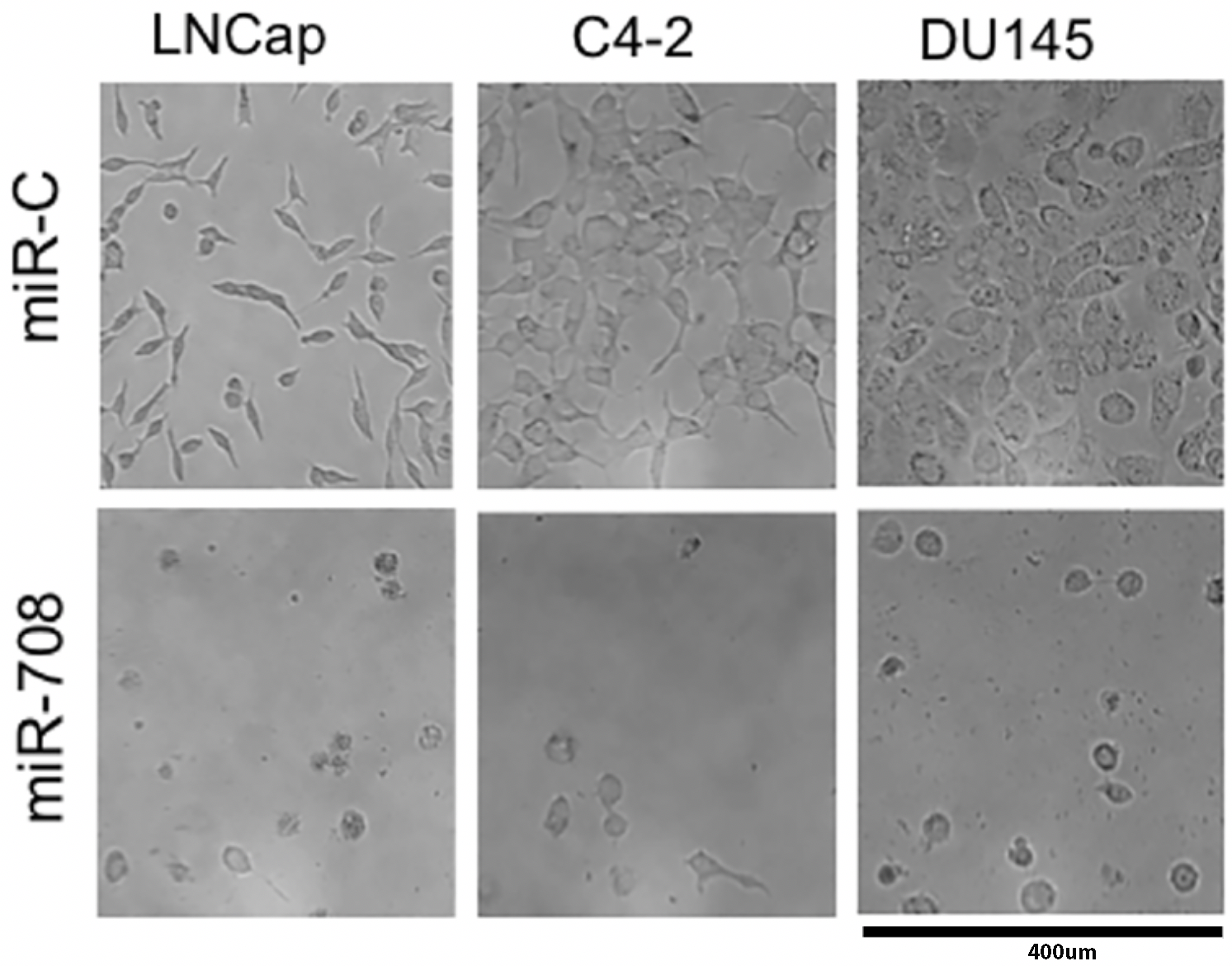


**Supplementary Figure 2**. LNCaP, C4-2, and DU145 cells were transfected with either control miRNA (miR-C) or miR-708. (**a**) miR-708 levels were quantified by RT-PCR. (**b**) Cell images were taken 24 h after transfection.


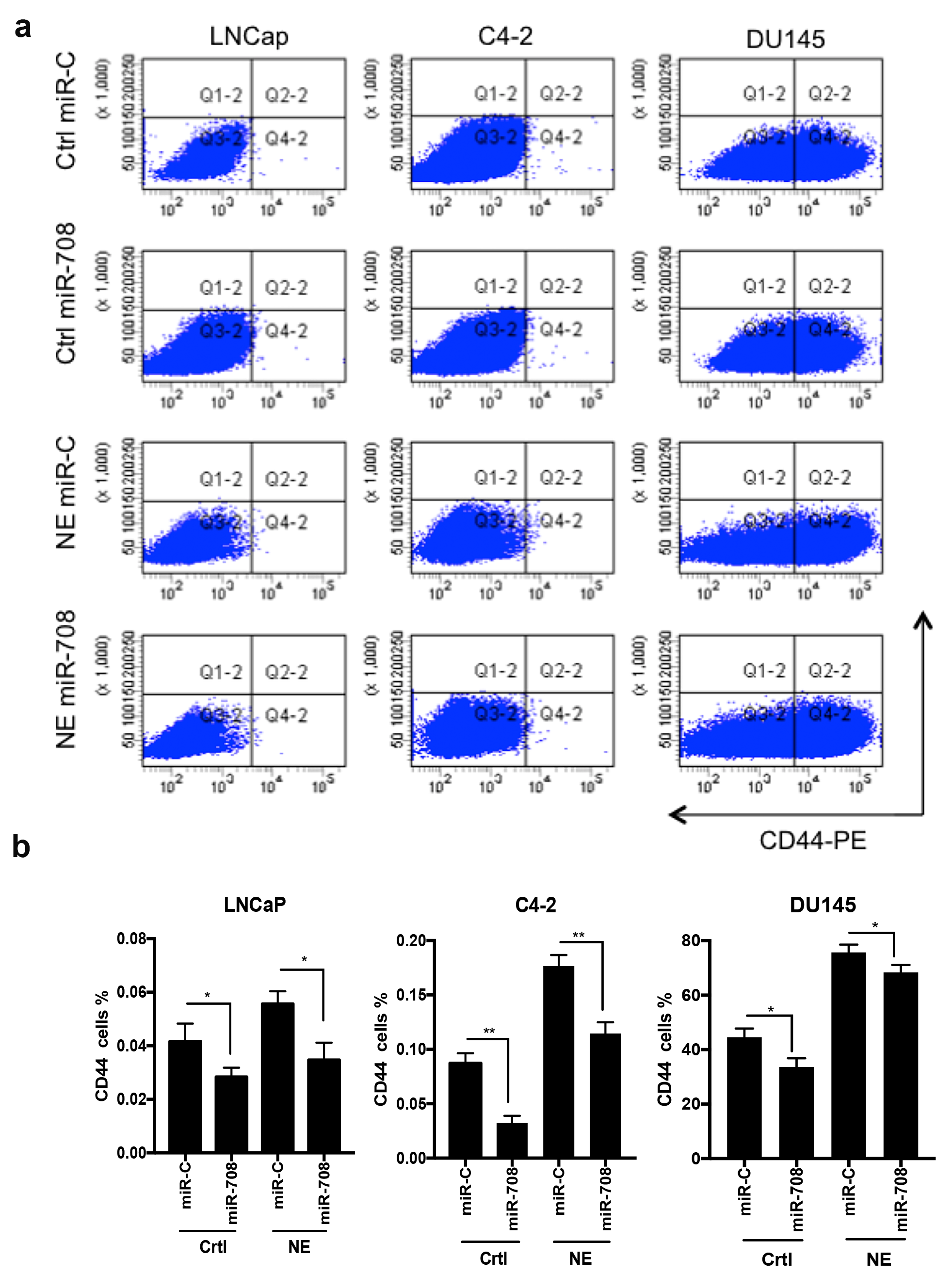


SSC-H

**Supplementary Figure 3**. Ectopic-expression of miR-708 reduces the frequency of CD44 expressing PC cells. Control (Crtl) or NE cells were transfected with either miR-C or miR-708 and followed by flow cytometry analysis. (**a**) Example of flow cytometry image. (**b**) The CD44 percentage diagram of 3 independent experiments.


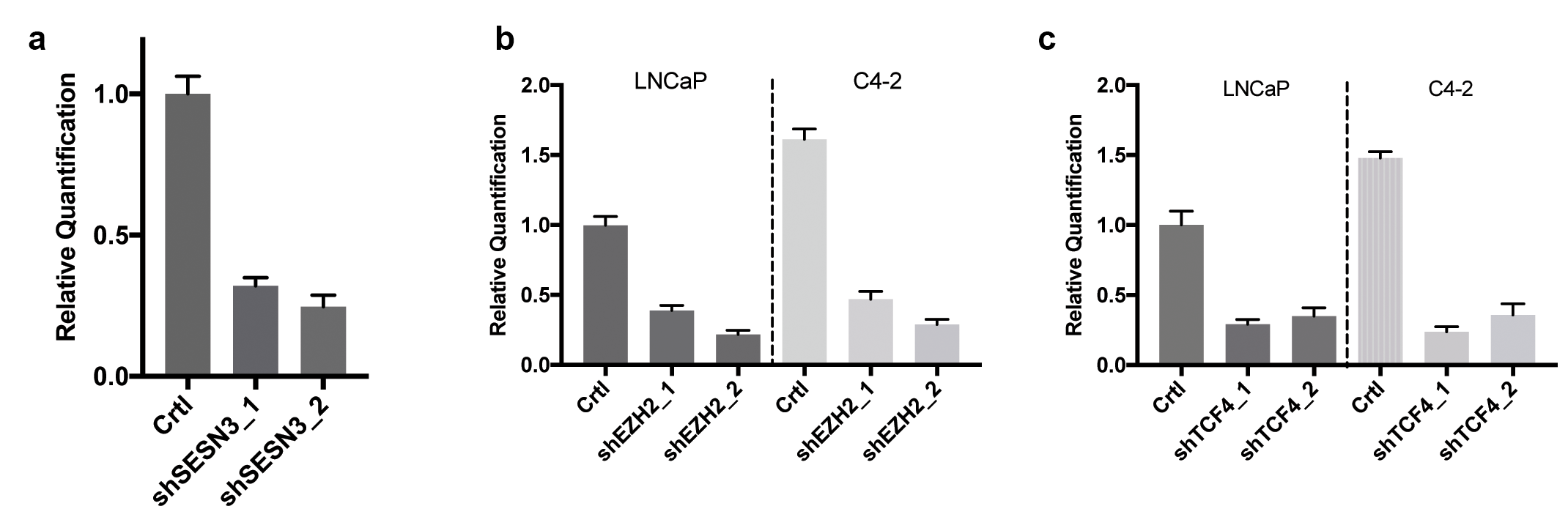


**Supplementary Figure 4**. qPCR analysis of (a) SESN3 in LNCaP cells, (b) EZH2 and (C)TCF4 expression in PC cells LNCaP and C4-2 and their derived cells with stably expressed shRNA.


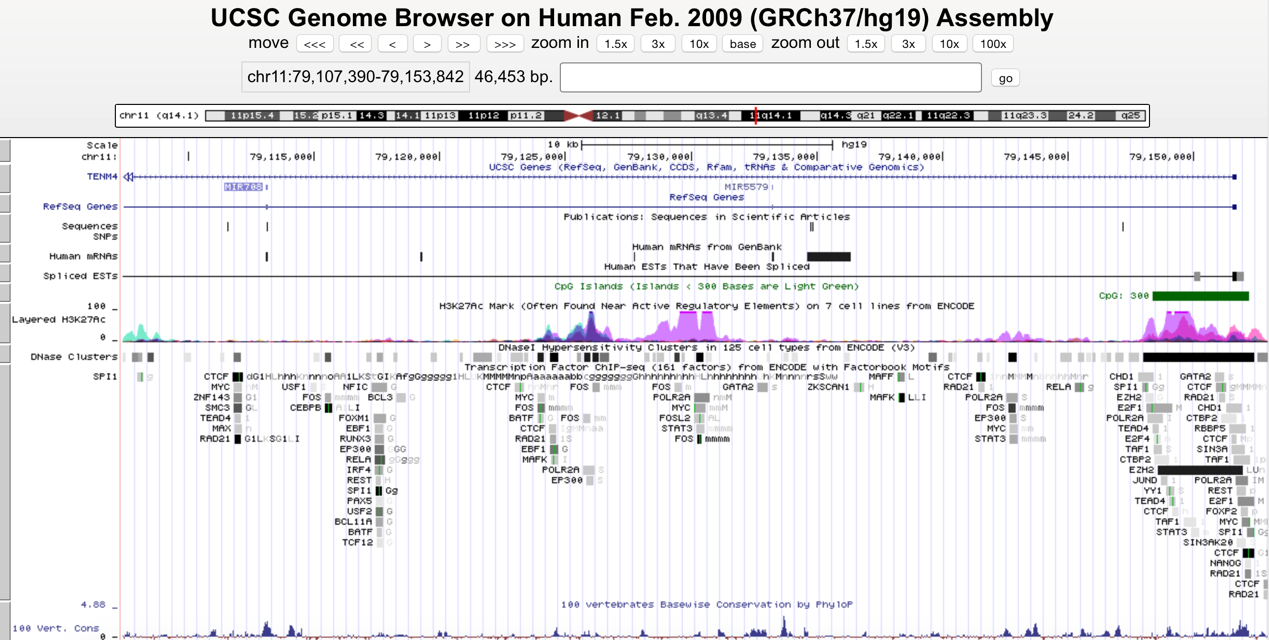


**Supplementary Figure 5**. UCSC genome browser snapshot of miR-708 promoter region.

**Supplementary Table 1.** **The sequences of oligos**

| Gene or site | Experiment | Sequence |
| --- | --- | --- |
| EZH1 | qPCR | Forward: GCTTCCTTCACCCTTTTCATGC |
|  |  | Reverse: CGACGACCAGAGCACTTGGAG |
| EZH2 | qPCR | Forward: AGGACGGCTCCTCTAACCAT |
|  |  | Reverse: CTTGGTGTTGCACTGTGCTT |
| SUZ12 | qPCR | Forward: TGCAGTTCACTCTTCGTTG |
|  |  | Reverse: GAACCAGGCTTGTTTTCCTG |
| PHF19 | qPCR | Forward: CTACCTCGGGAAGATCAAGA |
|  |  | Reverse: CTAGGCAGATGTTGCACTTGG |
| TCF4 | qPCR | Forward: TGGAGGGCTCTTTAAGGGG |
|  |  | Reverse: GATCCGTTGGGGAGGTAGG |
| SESN3 | qPCR | Forward: CAGGCAGCAACTTTGGGATT |
|  |  | Reverse: GACGCCTCTTCATCTTCCCT |
| NSE | qPCR | Forward: CCGGGAACTCAGACCTCATC |
|  |  | Reverse: CTCTGCACCTAGTCGCATGG |
| CgA | qPCR | Forward: GCGGTGGAAGAGCCATCAT |
|  |  | Reverse: TCTGTGGCTTCACCACTTTTCTC |
| SCG3 | qPCR | Forward: GTCTTCATCAACTAGACGGGACT |
|  |  | Reverse: ACAATCTTGTCAAACACGGCTC |
| 5' EZH2 binding region of miR708 | ChIP-qPCR | Forward: AGAGCCAGCGTTCTACTC |
|  |  | Reverse: ACGTGTTTAGAAACCTGCC |
| shEZH2 targeting site 1 | shRNA | GAGGGAAAGTGTATGATAA |
| shEZH2 targeting site 2 | shRNA | GAAAGAACGGAAATCTTAA |
| shTCF4 targeting site 1 | shRNA | GGCACACATTGTCTCTAACAAA |
| shTCF4 targeting site 2 | shRNA | TAGCTGAGTGCACGTTGAAAG |
| shSESN3 targeting site 1 | shRNA | GACGAGGAGAAGAGCATTT |
| shSESN3 targeting site 2 | shRNA | CCAGAGAGAGATCCAGAAA |
| 3' UTR of SESN3, predicted miR-708 targeting site 1 | Luciferase assay | TATAAGAATCCTATAAAATTTGAACCAGAGCTCCTATTTAGTTGTTATAATG |
| 3' UTR of SESN3, predicted miR-708 targeting site 2 | Luciferase assay | AGTAGCACAATTCAACAGCAGGCTGAAAGTCGTGGCTCCTCTGCTCAGATTGAG |
| 3' UTR of SESN3, predicted miR-708 targeting site 3 | Luciferase assay | GTCCACTTGCAACATGGCCTTGCTACTTGGATTAGCTCCTTTAAGCCTGAAAAT |
